# Supplementary figures and images for: Impact of seasonal variation on the oral and nasopharyngeal microbiome in school-aged children: the school MicroBE initiative
Source: mSystems. 2025 Aug 8;10(9):e00467-25. doi: 10.1128/msystems.00467-25 (PMC12455914; doi:10.1128/msystems.00467-25)

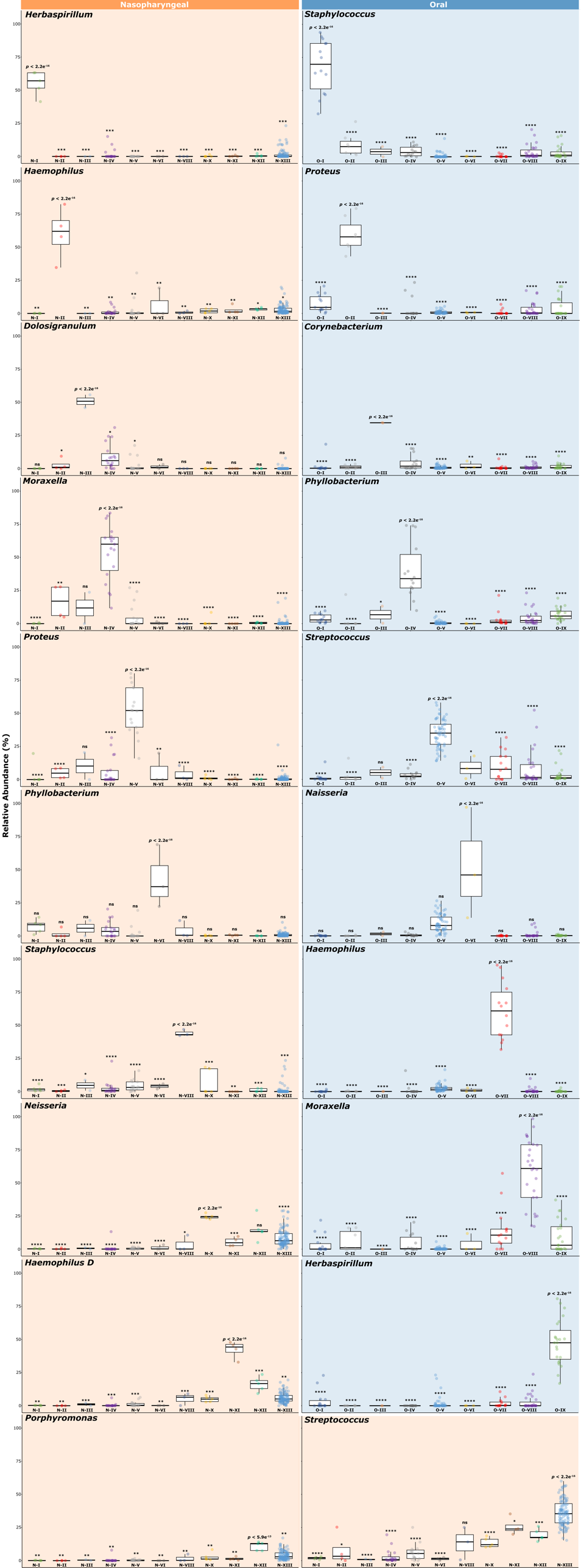

Supplement: Figure S1 — Boxplot comparing the relative abundances of the most abundant genera in oral (blue) and nasopharyngeal (orange) samples across three sampling times per group for each defined cluster. [file msystems.00467-25-s0001.pdf]
